# Increased mortality in women: sex differences in burn outcomes

**DOI:** 10.1186/s41038-017-0083-y

**Published:** 2017-06-04

**Authors:** Karen Karimi, Iris Faraklas, Giavonni Lewis, Daniel Ha, Bridget Walker, Yan Zhai, Gareth Graves, Sharmila Dissanaike

**Affiliations:** 1 Department of Surgery, Texas Tech University Health Sciences Center, 3601 4th Street MS 8312, Lubbock, TX 79430 USA; 2 Department of Surgery, University of Utah Health Sciences Center, 30 N 1900 East, Salt Lake City, UT 84132 USA

**Keywords:** Mortality, Sex, Gender, Burn, Survival, Outcome

## Abstract

**Background:**

There is increasing evidence that sex differences may influence responses after thermal injury and affect clinical outcomes. The objective of this study was to evaluate the relationships between sex, thermal injury, body size, and inpatient mortality in burn patients.

**Methods:**

Medical records of adults with >20% total body surface area (TBSA) burn injury admitted to two American Burn Association (ABA)-verified burn centers between 2008 and 2014 were retrospectively reviewed. Injury details and baseline characteristics, including body size as estimated by body surface area (BSA) and body mass index (BMI) were recorded, along with details of the hospital course. The primary outcome of inpatient mortality was compared between sexes.

**Results:**

Out of 334 subjects, 60 were women (18%). Median TBSA was 33% (IQR 25–49) in this cohort, with 19% full thickness burns and 30% inhalation injury. Despite no significant difference in age, presence of inhalation injury, TBSA, or depth of burn, women had significantly higher rates of inpatient mortality (45 vs. 29%, *P* = 0.01). BSA was significantly lower in women vs. men (*P* < 0.001), but this difference was not more pronounced among non-survivors. There was no difference in BMI between men and women non-survivors. Although not significant (*P* = 0.28), women succumbed to their injuries sooner than men (day 4 vs. 10 post-injury).

**Conclusions:**

Women are less likely to survive burn injuries and die sooner than men with similar injuries. Body size does not appear to modulate this effect. Burn centers should be aware of the higher mortality risk in women with large burns.

## Background

Burn size, age of the patient, and presence of inhalation injury are the primary factors that contribute to mortality in burn patients [1, 2]. Scoring systems such as the Baux rule and its subsequent revisions have been developed to estimate the likelihood of death after burn injury using these parameters and are in common use in burn centers throughout the world [3, 4]. Sex of the patient is not routinely included in these determinations and historically has not been considered a major factor in influencing the outcomes of burn injury. However, there is increasing evidence that sex differences may influence responses to injury in other fields. For example, several studies support improved outcomes for women sustaining traumatic injury [5, 6]. In traumatic brain injury, female sex is associated with higher mortality, particularly in post-menopausal women, suggesting a possible hormonal influence [7]. Sex dimorphism has also been evaluated in other acute illnesses such as post-surgical infections and sepsis [8–11]. It is plausible that these findings of sex differences may hold true in burn injury as well. In an era where personalized medicine is increasingly important, investigating differences based on sex and gender are important in all fields, including injury.

The potential for sex differences to affect outcomes following burn injury has not been well explored, and the existing literature is somewhat contradictory. Several studies suggest that women who sustain burn injuries have an increased risk of death in comparison to men [3, 4, 12, 13]. Many of these studies indicate that sex differences were not found among the older age groups, aged 60 or above, suggesting possible hormonal influences on outcome. It has also been reported in some series that men have a significantly higher mortality rate following thermal injury than women [14], while other reports indicate no differences in outcomes between sexes [15, 16].

In this study, we sought to assess the relationship between sex and outcomes following burn injuries and to assess a possible impact of obesity on these outcomes.

## Methods

### Setting and data collection

An Institutional Review Board (IRB)-approved retrospective study was conducted at two American Burn Association (ABA)-verified burn centers in geographically diverse locations. Included in this study were adults >18 years of age, with >20% total body surface area (TBSA) burns, admitted between January 2008 and December 2014.

Study data were collected and managed using Research Electronic Data Capture (REDCap), an electronic data capture tool. REDCap is a secure, web-based application designed to support data capture for research studies, providing (1) an intuitive interface for validated data entry; (2) audit trails for tracking data manipulation and export procedures; (3) automated export procedures for seamless data downloads to common statistical packages; and (4) procedures for importing data from external sources [17].

### Study variables

Variables collected include basic demographics, injury characteristics, comorbidities, operations performed, hospital course, complications, clinical outcomes including inpatient mortality and time until healed, and discharge disposition. Comorbid conditions were initially abstracted from the national ABA/TRACS (Trauma Registry of the American College of Surgeons) registry data using definitions based on the ABA National Burn Data Standard Dictionary [18] and subsequently verified by chart review of medical record documentation. Missing data on comorbidities at the time of admission was assumed to be normal. Sequential Organ Failure Assessment (SOFA) II scores were based on abstracted parameters from the first and second 24 h post-admission. Obesity was estimated using both body mass index (BMI) and Mosteller body surface area (BSA). The number of days for wound to be healed was the difference between date of injury and clinic date that wound was closed.

### Statistical analysis

We used STATA 13.0 (College Station, TX) for all analyses. A two-sided alpha of 0.05 was considered as the threshold for statistical significance. Values were reported as a median with interquartile range (IQR) or percentage unless otherwise stated. Chi-squared or Fisher’s exact was used for categorical or nominal variables. For continuous variables, rank-sum test was used when comparing sex. To estimate survival time, Kaplan-Meier method and risk table were included. Patients who had a length of stay (LOS) greater than 90 days (*N* = 18) had LOS censored at 90 days in the Kaplan-Meier figure to prevent a misleading estimate with respect to sample size. Multivariable analysis was performed; however, the models were limited in validity for predicting the effect of sex on outcomes by the relatively small number of women in the study, collinearity between variables and the strong predictive value of TBSA and age superseding other variables (data not shown).

## Results

### Demographics

A total of 334 patients that fit the inclusion criteria were admitted during the review period, and their records were obtained and analyzed. Patient’s demographics, injury etiology (Table 1), and interventions (Table 2) were compared by sex. The sample included 60 (18%) women. The median TBSA of burns was 34% (IQR 25–50). There were no significant differences between men and women in terms of TBSA (37 vs. 33%, *P* = 0.34), proportion of full-thickness burn (21 vs. 18%, *P* = 0.17), proportion of partial thickness burn (14 vs. 17%, *P* = 0.26), or inhalation injury (38 vs. 28%, *P* = 0.18). Comorbidities among men and women were statistically similar; however, women were less likely to suffer from alcoholism compared to men (5 vs. 16% respectively, *P* = 0.02).

**Table 1:** Demographics and injury by sex (*N* = 334)

Variable	*P* value	Total *N* = 334	Female *N* = 60	Male *N* = 274
Age	0.45	43 (30–55)	45 (33–59)	43 (30–55)
Caucasian^a^	0.39	271/286 (95%)	48/50 (96%)	223/236 (94%)
Hispanic/Latino^a^	0.14	87/282 (31%)	11/50 (22%)	76/232 (33%)
Body mass index	0.31	28.2 (24.4–32.4)	27.8 (23.0–32.9)	28.3 (24.8–32.4)
Body surface area	<0.001	2.03 (1.88–2.25)	1.86 (1.68–2.0)	2.09(1.92–2.27)
At least one comorbidity	0.13	182 (55%)	38 (63%)	144 (53%)
Diabetes mellitus	0.10	25 (7%)	8 (13%)	17 (6%)
Current smoker	0.99	78 (23%)	14 (23%)	64 (23%)
Chronic drinker	<0.05	48 (14%)	3 (5%)	45 (16%)
Drug addict	0.99	28 (8%)	5 (8%)	23 (8%)
Hypertension requiring medication	0.72	56 (17%)	11 (18%)	45 (16%)
Mental health diagnosis	0.18	38 (11%)	10 (17%)	28 (10%)
% TBSA	0.34	33% (25–49%)	37% (25–60%)	33% (25–48%)
% Full thickness	0.17	19% (4–37%)	21% (4–47%)	18% (4–34%)
% Partial thickness	0.26	17% (6–26%)	14% (3–26%)	17% (7–25%)
Full thickness/TBSA ratio	0.24	0.56 (0.41−0.89)	0.76 (0.18−.94)	0.55 (0.13−0.88)
Inhalation injury	0.12	100 (30%)	23 (38%)	77 (28%)
Revised Baux	0.16	85 (66−109)	91 (66−125)	85 (66−107)
Hematocrit	<0.001	47 (43–52)	44 (41–49)	48 (44–53)
Worst base deficit in 1st 24 h	0.07	−6.4 (−10.1, −3.4)	−8.3 (−11.6, −4.4)	−6 (−9.9, −3.2)
Worst base deficit in 2nd 24 h	0.82	−4.7 (−7.3, −2.4)	−4.6 (−7.9, −2)	−4.7 (−7.2, −2.4)
BUN	<0.05	14 (10–17)	13 (9–17)	14 (11–17)
Admission blood glucose	0.55	137 (113–179)	142 (117–186)	135 (112–178)
Admit HgA1c	0.58	5.5 (5.3–6.2)	5.8 (5.3–6.5)	5.5 (5.3–6.2)
Admit albumin	0.05	3.2 (2.5–3.6)	2.9 (2.4–3.3)	3.3 (2.5–3.7)
SOFA in 1st 24 h	0.80	6 (2–8)	6 (2–8)	6 (3–8)
SOFA in 1st 24–48 h	0.70	6 (2–8)	3 (1–7)	3 (1–5)

*TBSA* total body surface area, *BUN* blood urea nitrogen, *SOFA* sequential organ failure assessment

Values reported as median (interquartile range) or *n* (%) unless otherwise stated; *P* values: comparing sites using Rank-sum test for continuous or chi-squared or Fisher’s exact for nominal or categorical variables

^a^Unknown were excluded

Regarding obesity, the median BMI was 28.2 (IQR 24–32). While BMI did not significantly differ between the sexes (*P* = 0.31), BSA was significantly lower in women (*P* < 0.001) in both survivor and non-survivor groups.

Women had a significantly lower hematocrit (*P* < 0.001) and blood urea nitrogen (BUN) levels (*P* = 0.04) upon admission than men; however, these differences disappeared in the non-survivor group. Women also had significantly lower BUN levels early in the clinical course compared with men.

### Surgical intervention

There was no difference in time to first operation between men and women, occurring at a median of 3 days (*P* = 0.42) (Table 2). There were also no differences between men and women in other parameters of care delivery. The surgical outcomes did not significantly differ between the sexes except that women were more likely to experience urinary tract infection (18 vs. 9%, *P* = 0.04). Lengths of stay were not statistically different.

**Table 2:** Intervention and outcomes by sex (*N* = 334)

Variable	*P* value	Total *N* = 334	Female *N* = 60	Male *N* = 274
Days from admit to 1st operation	0.42	3 (1–5)	3 (1–7)	3 (1–5)
Operation total	0.66	3 (1–6)	2 (16)	3 (1–16)
Postoperative complication	0.76	133 (40%)	22 (37%)	111 (41%)
Requiring autograft	0.08	241 (72%)	38 (63%)	203 (74%)
Autograft complication	1.00	53 (16%)	8 (13%)	45 (16%)
Requiring homograft	0.28	62 (19%)	8 (13%)	54 (20%)
Homograft complication	<0.05	23 (7%)	6 (10%)	17 (6%)
Integra	0.53	43 (13%)	6 (10%)	37 (14%)
Integra complication	0.66	16 (5%)	3 (5%)	13 (5%)
Patients requiring intubation	0.86	258 (77%)	47 (78%)	211 (77%)
Ventilator days	0.73	3 (1–14)	3 (1–11)	3 (1–15)
Length of stay	0.61	28 (18–45)	27 (18–48)	28 (18–43)
Length of stay per TBSA^a^	0.40	0.89 (0.67–1.33)	0.991 (0.68–1.58)	0.89 (0.66–1.29)
Readmission^a^	0.41	65 (19%)	7 (12%)	58 (21%)
Inpatient mortality	<0.05	107 (32%)	27 (45%)	80 (29%)

*TBSA* total body surface area

Values reported as median (interquartile range) or *n* (%) unless otherwise stated; *P* values: comparing sites using Rank-sum test for continuous or chi-squared or Fisher’s exact for nominal or categorical variables

^a^Survivors only

### Inpatient mortality

Women had a significantly higher risk of inpatient mortality than men (45 vs. 29%, *P* = 0.02). Despite a higher risk of inpatient mortality, women had similar demographics and etiology among non-survivors including TBSA (53 vs. 59%, *P* = 0.66), proportion of full-thickness burn (*P* = 0.77), age (*P* = 0.10), and other demographics as shown in Table 3. Female non-survivors were older than the men who died; however, this difference was not statistically significant (*P* = 0.10). Among the non-survivors, BMI was not significantly different (*P* = 0.46). BSA was significantly lower in women (*P* = 0.001), which was similar to findings in survivors. Comorbidities among non-surviving men and women were statistically similar.

**Table 3:** Non-survivors only: demographics and injury by sex (*N* = 107)

Variable	*P* value	Total *N* = 107	Female *N* = 27	Male *N* = 80
Age	0.10	55 (40–69)	63 (45–73)	53 (39–66)
Caucasian^a^	0.19	77/83 (93%)	17/19 (89%)	60/64 (94%)
Hispanic/Latino^a^	<0.05	31/81 (38%)	3/19 (16%)	28/62 (45%)
Body mass index	0.46	27.9 (24.0–32.4)	27.1(22.3–32.9)	28.0 (24.5–32.2)
Body surface area	<0.05	2.01 (1.86–2.21)	1.87 (1.56–2.1)	2.06(1.90–2.20)
At least one comorbidity	0.06	67 (63%)	21 (78%)	46 (58%)
Diabetes mellitus	0.14	11 (10%)	5 (19%)	6 (8%)
Current smoker	0.40	21 (20%)	7 (26%)	14 (18%)
Chronic drinker	0.15	19 (18%)	2 (7%)	17 (21%)
Drug addict	0.64	6 (6%)	2 (7%)	4 (5%)
Hypertension requiring medication	<0.05	24 (22%)	8 (30%)	16 (20%)
Mental health diagnosis	0.34	14 (13%)	5 (19%)	9 (11%)
% TBSA	0.66	58 (36–77)	53 (36–70)	59 (35–82)
% Full thickness	0.77	46 (22–70)	46 (24–67)	43 (21–73)
% Partial thickness	0.36	7 (3–20)	6 (3–13)	8 (2–22)
Full thickness/TBSA ratio	0.71	0.90 (0.67–0.99)	0.92 (.75–.99)	0.89 (.56–1.00)
Inhalation injury	0.12	53 (50%)	17 (63%)	36 (45%)
Revised Baux	0.17	122 (101–139)	127 (117–141)	115 (101–139)
Hematocrit	0.09	46 (41–53)	44 (40–48)	47 (41–55)
Worst base deficit in 1st 24 h	0.49	−9.8 (−12.8, −6.2)	−9.4 (−12.2, −7.1)	−9.8 (−14.6, −6)
Worst base deficit in 2nd 24 h	0.24	−6.9 (−9.5, −3.9)	−6.3 (−8.5, −3.2)	−6.9 (−10.4, −3.9)
BUN	0.74	16 (12–18)	16 (9–18)	16 (12–18)
Admission blood glucose	0.32	167 (129–226)	181 (138–235)	164 (127–220)
Admit HgA1c	0.62	5.8 (5.2–6.5)	6.1 (5.2–9.9)	5.5 (5.2–6.4)
Admit albumin	0.83	2.8 (2.1–3.2)	2.8 (2.2–3.2)	2.8 (2.1–3.3)
SOFA in 1st 24 h	0.99	8 (6–10)	8 (6–10)	8 (6–10)
SOFA in 1st 24–48 h	0.55	8 (6–10)	9 (6–11)	8 (5–10)

*TBSA* total body surface area, *BUN* blood urea nitrogen, *SOFA* sequential organ failure assessment

Values reported as median (interquartile range) or *n* (%) unless otherwise stated; *P* values: comparing sites using Rank-sum test for continuous or chi-squared or Fisher’s exact for nominal or categorical variables

^a^Unknown were excluded

**Fig. 1:**
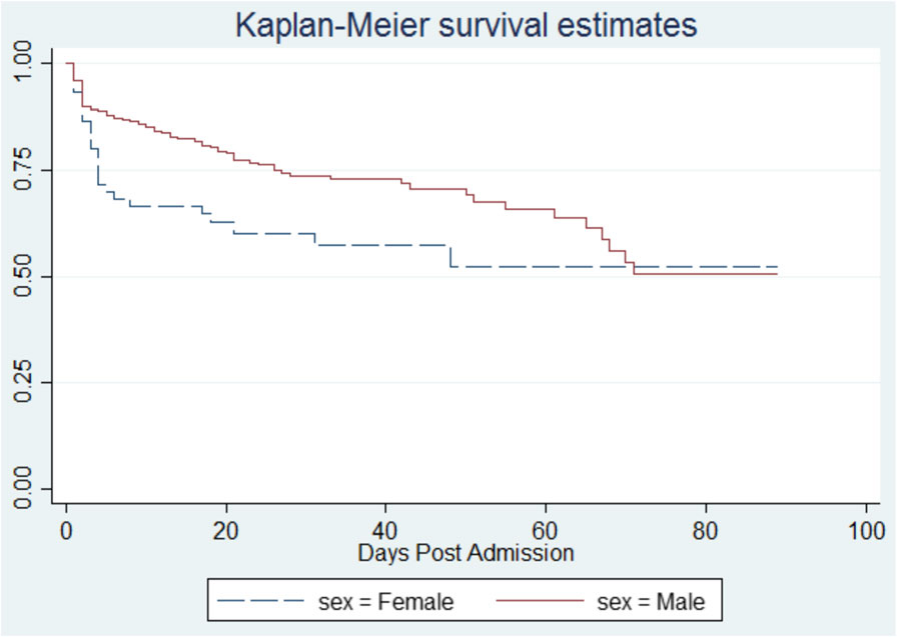
Kaplan-Meier survival curve. Women trended toward earlier deaths on day 4 (IQR 2–17) vs. day 10 (IQR 2–24); however, this trend was not found to be statistically significant (*P* = 0.28)

Additionally, death occurred much sooner in women compared to men (4 vs. 10 days, *P* = 0.28). The Kaplan-Meier survival curve (Fig. 1) shows a dramatic drop in women compared to men during the first 30 days post-injury; however, this survival estimate decreases as patients stay in the hospital past 30 days. Despite women trending toward earlier deaths on day 4 (IQR 2–17) vs. day 10 (IQR 2–24), the trend was not found to be statistically significant (*P* = 0.28). There was no significant difference in outcomes between institutions.

## Discussion

The findings of this study suggest that women who sustain a burn injury are less likely to survive compared to men. Body size does not appear to modulate this effect, as BMI and BSA did not differ among survivors and non-survivors in either sex. Our results are similar to several other published reports indicating that women are more likely than men to die after burn injury [3, 4, 12, 13, 19, 20].

O’Keefe et al. showed the risk of death for women between the ages of 30 and 59 to be nearly twice that in men of the same age [21]. The differing case fatality rates could not be attributed to any imbalance in risk factors, such as burn size or depth, inhalation injury, or age distribution within the 30- to 59-age bracket, or to any differences in complication incidence, such as acute respiratory distress syndrome (ARDS), sepsis, acute renal failure, or pneumonia. No sex-related differences in mortality were found in the younger or older age groups in the same study. Likewise, Kerby et al. found that women between the ages of 20 and 69 sustaining thermal injury have higher odds of mortality when compared with men; however, no sex differential in mortality odds were found in the younger or older age groups [13]. These differences could not be explained by differences in age, race, TBSA burn, inhalation injury, or the presence of pneumonia. McGwin et al. came to a similar conclusion suggesting that there was no sex differential in mortality risk for burn patients 60 years and older [12].

In modern series, infection and sepsis are the primary reasons for mortality occurring after the initial resuscitation period. Therefore, differential response to infection may be a factor in explaining this sex disparity in survival. Differences in infections and immune response between sexes after a burn injury have been described in the literature. Gregory et al. demonstrated a sex difference in cell-mediated immune responses and interleukin (IL)-6 production after thermal injury. After injury, male mice have a rapid increase in IL-6 production and corresponding suppression of cell-mediated immunity, delayed-type hypersensitivity, and splenocyte proliferative responses within the first 24 h, whereas suppression of cell-mediated immunity in female mice is delayed until day 10, as IL-6 levels increase steadily over time [22]. These differences in immune response may explain the increased risk for women in developing infectious complications that lead to sepsis, multiple-system organ failure, and death [13]. Furthermore, the temporal relationship between post-burn time and immune response may explain the differences in the timing of death between men and women.

There are conflicting reports on the association between sex and survival after sepsis in other fields [9, 23, 24]. Several studies have shown that women tolerate infectious challenge better than men [10, 25] and are less likely to die from sepsis, suggesting a sex difference in the immune response [9]. In response to endotoxemia, van Eijk et al. found that women show a more pronounced innate pro-inflammatory immune response with increased rises in C-reactive protein and tumor necrosis factor-α and less attenuation of norepinephrine sensitivity compared to men [26]. Horton et al. examined sex-related differences in post-burn myocardial inflammation and discovered female burns elicited lower IL-10 levels compared to male burns, correlating with a reduced pro-inflammatory response and a subsequently reduced need for compensatory anti-inflammatory response [27]. Offner et al. show that men have an increased risk of developing major infections following severe injury compared to women; however, mortality rates were found to be similar across both sexes [8]. Pietropaoli et al. and McGwin et al. posit that the female sex is an independent risk factor for mortality in critically ill surgical patients with sepsis [11, 23]. It has also been reported that the female sex is at increased risk for death following hospital-acquired pneumonia [24].

The effects of sex hormones on differences in clinical outcomes have been suggested previously, and explored in a few studies. Increases in IL-6 in female mice following burn injury have been shown to correlate with increased circulating estrogen (E2) levels [28]. Onset of critical illness or trauma has been shown to alter the production of gonadal sex hormones, and circulating concentrations of E2 are enhanced within the first few days following sepsis or thermal injury [29–31]. It is particularly noteworthy that the increased likelihood of death among female burn patients has only been apparent during the reproductive years, according to several studies [12, 13, 21]. It is suggested that the lack of sex differences in mortality among the younger and older age groups reflects the decreased levels of estrogen in younger women and post-menopausal women, negating the increased risk of mortality following thermal injury [13]. However, in the current study, the mean age of non-survivor women was 63 years, with the first quartile at 45 years. In comparison, the median age of male non-survivors was 53 years with the first quartile at 39 years. Therefore, male non-survivors trended younger than their female counterparts and there was no discernible adverse effect on survival of being a premenopausal woman. Jeschke et al. also posit that women exert an attenuated inflammatory and hypermetabolic response to a severe burn compared to men, as female pediatric patients in their study showed higher levels of endogenous anabolic hormones, higher estrogen, attenuated stress hormones, and attenuated inflammatory markers [16]. This decrease is reflected in enhanced muscle protein balance and preservation of lean body mass, which are associated with significantly shortened hospital stays. The results of the current study showed a significantly decreased BUN in women on admission, although this difference disappeared in non-survivors. This further adds to the hypothesis that sex differences in protein catabolism may play a role in burn outcome. Angstwurm et al. suggest that mortality is not dependent on gender but rather correlated with elevated sex hormones across both genders: 17beta-estradiol and testosterone in women and 17beta-estradiol and progesterone in men [32].

Another posited explanation for sex differences in burn outcome relies on the etiology or type of burn being the important confounding variable in the relationship between sex and mortality. For example, Muller et al. described a series in which the majority of female burns were self-inflicted; since self inflicted burns tend to be deeper and more extensive, survival is less likely [3]. Fowler et al. suggest that differences in admission practices, decision-making, and processes of care influencing clinical outcomes for critically ill patients may be a cause for older women having a greater risk of death in the intensive care unit (ICU) and hospital after critical illness [33]. Pietropaoli et al. confirm significant disparities in some aspects of care delivery in their study; women were more likely to have limitations placed on aggressive interventions including resuscitation in case of cardiopulmonary arrest and receive packed red blood cell transfusions while men were more likely to receive invasive mechanical ventilation at admission, deep venous thrombosis prophylaxis, and hemodialysis catheters. These differences, however, could not explain the 10% higher mortality risk in female patients with severe sepsis or septic shock [11]. In the present study, we did not see differences in time to excision and grafting or other parameters of care delivery, suggesting this is less likely to be a source of the differential survival.

Differences in body fat distribution between sexes may be a potential explanation for survival differences. Body fat distribution between sexes and across ethnicities has been studied and found to have consistent patterns. Men are more likely to distribute fat in the central or abdominal fat depots and women in more peripheral areas such as gluteal and femoral subcutaneous fat depots. Lipoprotein A lipase activity was increased in omental and visceral fat but not in subcutaneous fat, implying differential sex metabolic activity based on fat distribution [34–36]. Obesity did not seem to impact overall distribution of fat but showed an increase in fat cell size [35, 36]. While many suggest that body fat distribution and hormone-related receptors are important predictors of fat metabolism and related morbidity and mortality, no clear links or mechanisms have been offered in current literature. Another potential explanation for survival differences between sexes is related to differential activity of brown fat activation and metabolism in relation to hormone secretion between pre-menopausal women and men; however, this is not very well understood and speculative [37].

Limitations of the current study include its limited size and retrospective design. The small sample size decreases statistical power and increases the probability of a type II error where we may have not found statistical significance for outcomes differences between sexes that exist. The small number of women in this study (stemming from the preponderance of male burn patients in technologically advanced countries) precluded any multivariable analysis. Given the disproportionate impact of TBSA, age, and inhalation injury on burn outcome, having only 60 women is too small a study population to perform a meaningful multivariable analysis of the impact of sex on outcome. In addition, the retrospective nature of the study, combined with the lack of information on menopausal symptoms in routine admission documentation for burns, did not allow meaningful analysis of differences in outcomes in pre- vs. post-menopausal women, which is an avenue that deserves further exploration. Further analyses should also be conducted regarding the differences in sepsis and organ complications between men and women following thermal injury. Finally, the lack of outcome difference based on BSA and BMI could mean either that no survival differences based on body fat composition exist or that these variables are not sensitive enough to detect a real difference. As such, we suggest that future studies on body fat composition use bioimpedence analysis or dual-energy X-ray absorptiometry (DEXA) for greater accuracy, instead of estimations such as BMI or BSA.

**Fig. 2:**
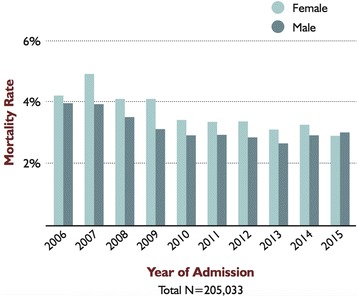
Mortality rate by gender, 2006–2015. Figure reproduced from the National Burn Repository [38] with permission from the American Burn Association

We present these results as hypothesis-generating rather than a conclusive statement on sex and burn outcomes; studies of larger populations are needed, although recent data from the National Burn Repository support our findings of a subtle trend toward higher mortality in women nationwide (Fig. 2). Based on the trend toward earlier mortality in women, it might be beneficial to evaluate differences in responsiveness to resuscitation between sexes, in addition to investigating cytokines and other hormonal differences that may reveal biochemical reasons underlying the observation of poorer outcome.

## Conclusions

Sex-based differences in outcomes appear to exist within centers and across centers, suggesting that biologic factors rather than differences in clinical care account for these variations. Women are more likely to die compared to men after burn injury despite similar age and injury characteristics.

## Abbreviations

ABA: American Burn Association
ARDS: Acute respiratory distress syndrome
BMI: Body mass index
BSA: Body surface area
BUN: Blood urea nitrogen
DEXA: Dual-energy X-ray absorptiometry
ICU: Intensive care unit
IL: Interleukin
IQR: Interquartile range
IRB: Institutional Review Board
LOS: Length of stay
REDCap: Research Electronic Data Capture
SOFA: Sequential Organ Failure Assessment
TBSA: Total body surface area
TRACS: Trauma Registry of the American College of Surgeons
